# Caspase 2 Activation and ER Stress Drive Rapid Jurkat Cell Apoptosis by Clofibrate

**DOI:** 10.1371/journal.pone.0045327

**Published:** 2012-09-18

**Authors:** Fabio Penna, Fabrizio Pin, Domiziana Costamagna, Patrizia Reffo, Francesco Maria Baccino, Gabriella Bonelli, Paola Costelli

**Affiliations:** Department of Experimental Medicine and Oncology, University of Torino, Torino, Italy; University of Frankfurt - University Hospital Frankfurt, Germany

## Abstract

Differently from the antiapoptotic action most commonly assigned to peroxisome proliferators (PPs), we demonstrated that some of them, clofibrate (CF) in particular, display clearcut apoptogenic properties on rat hepatoma cell lines. We and others could confirm that CF as well as various other PPs can induce apoptosis in a variety of cells, including human liver, breast and lung cancer cell lines. The present study was aimed at investigating the cytotoxic action of CF on a neoplastic line of different origin, the human T leukemia Jurkat cells. We observed that CF rapidly triggers an extensive and morphologically typical apoptotic process on Jurkat cells, though not in primary T cells, which is completely prevented by the polycaspase inhibitor zVADfmk. Gene silencing studies demonstrated that CF-induced apoptosis in Jurkat cells is partially dependent on activation of caspase 2. Looking for a possible trigger of caspase 2 activation, we observed increased levels of phosphorylated eIF2α and JNK in CF-treated cells. Moreover, intracellular Ca^2+^ homeostasis was perturbed. Together, these findings are suggestive for the occurrence of ER stress, an event that is known to have the potential to activate caspase 2. The present observations demonstrate that CF induces in Jurkat cells a very fast and extensive apoptosis, that involves induction of ER stress and activation of caspases 2 and 3. Since apoptosis in Jurkat cells occurs at pharmacologically relevant concentrations of CF, the present findings encourage further in depth analysis in order to work out the potential implications of CF cytotoxcity on leukemic cells.

## Introduction

Clofibrate (CF) and other fibrate derivatives have long been used as hypolipidemic drugs [1]. These compounds are part of a largely heterogeneous class of chemicals known as peroxisome proliferators (PPs). Their mechanism of action typically requires binding to heterodimeric nuclear receptors in which a monomer of RXR combines with a monomer of PP-activated receptor (PPAR). Three different PPAR subfamilies (α, β/δ, and γ) have been described [2], PPARα being particularly involved in fibrate-activated signal transduction.

PPs have been shown to behave as hepatocarcinogens in rodents [3]. Indeed, when administered to rats and mice they induce peroxisome proliferation, hepatomegaly, and hepatocarcinogenesis [4], [5]. By contrast, these effects cannot be observed in monkeys, pigs and humans [6], [7], [8]. PPs are considered non-genotoxic carcinogens, their oncogenicity apparently deriving from both the oxidative response consequent to peroxisome proliferation and their ability to interfere with the regulation of cell proliferation and death [8], [9]. PPARα appears mainly in charge of these activities. Indeed, long term PPs administration does not result in hepatocarcinogenesis in PPARα-null mice [10]. However, several side effects such as rhabdomyolysis, liver and heart toxicity, anemia and leukopenia as well as rodent liver carcinogenesis are likely due to PPAR-independent mechanisms (rewieved in [11]). In addition, despite observations that various PPARα ligands exert a prosurvival action that was suggested to contribute to their carcinogenic potential [12], some of them have been demonstrated to induce apoptosis in different hepatoma cell lines.

An initial report from our laboratory [13] showed, quite unexpectedly at the time, that treatment with CF promptly induces massive and typical apoptosis in hepatoma cells of both rat (Yoshida AH-130) and human (HepG2) origin, with no correlation with the species-specificity of hepatocarcinogenesis. Subsequently, similar observations were made on various cell lines exposed to CF or other PPARα ligands such as nafenopin, perfluorooctanoic acid, and BR931 [14], [15], [16]. Noterworthily, PPARα ligand cytotoxicity is not restricted to cells of the hepatocytic lineage, but it has also been observed in breast or lung cancer cell lines [17], [18] as well as in human keratinocytes and lymphoblasts [19], [20]. Furthermore, ligands of the other two PPAR isotypes, *i.e.* β/δ, and γ, have been shown to induce cell death as well [21], [22], [23], and CF itself can bind to all three PPAR subfamilies [24].

**Figure 1:**
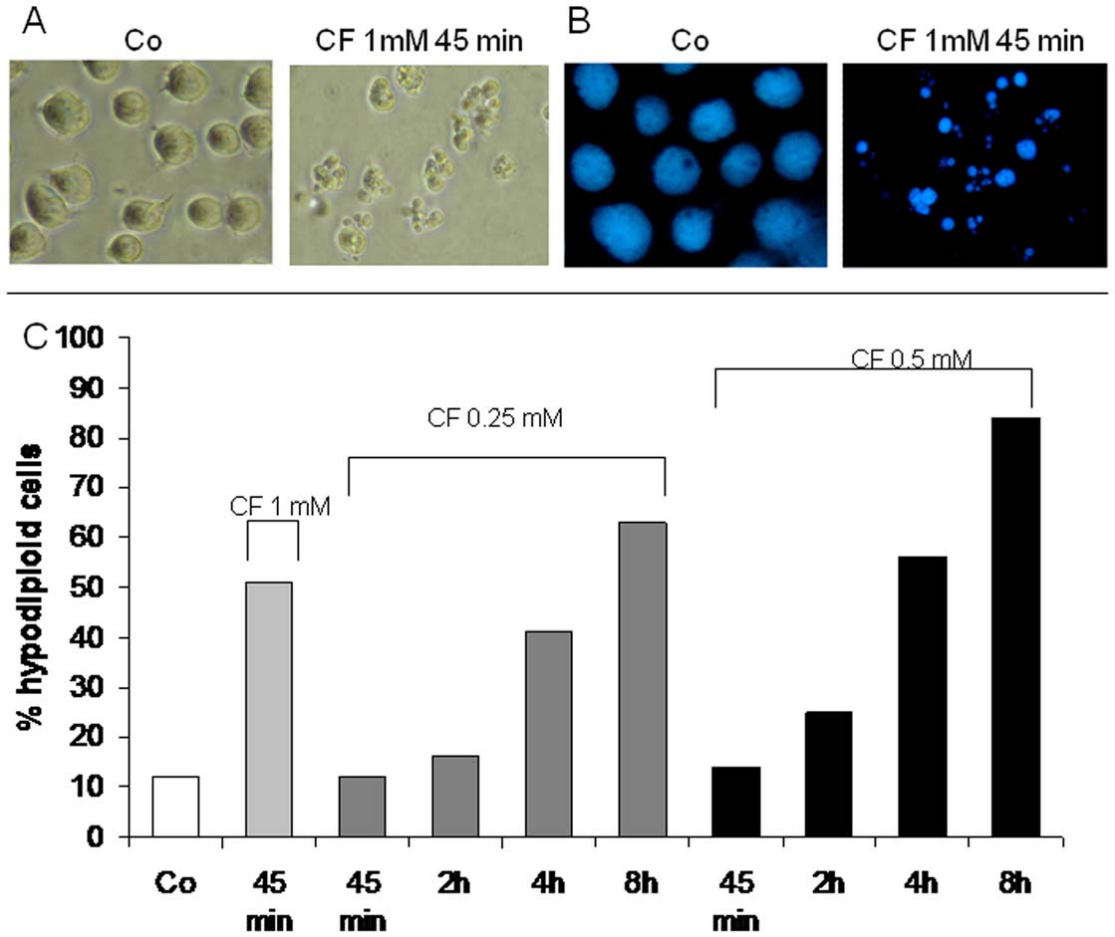
Kinetic of clofibrate-induced death in Jurkat cells. Morphological appearance (A: phase contrast microscopy, B: DAPI staining) of Jurkat cells exposed to clofibrate (CF) 1 mM for 45 min (Co: control). (C) percentages of cells with hypodiploid DNA content (apoptotic; see Penna et al., 2009) after exposure to CF (concentration on top of the bars, time on X axis). Data are expressed as mean ± SD (n = 3). ** p<0,01 vs Co; *** p<0,001 vs Co.

Of particular interest are several reports that suggest the potential use of PPARα ligands as antineoplastic drugs. In this connection, a good insight into cell death mechanisms triggered by these ligands becomes especially important. Previous results obtained in our laboratory suggested that a role may be played by inhibition of HMG-CoA reductase (HMGR), a key enzyme in isoprenoid biosynthesis. Indeed, the mRNA level and enzymatic activity of HGMR as well as the cholesterol content in mitochondria are reduced in Yoshida AH-130 cells soon after CF treatment, while cell death can be attenuated by supplementing cells with mevalonate, the reaction product of HMGR [25]. Quite recently we also demonstrated [26] that the rapid apoptosis induced by CF in the AH130 hepatoma cells is amenable to a classic caspase-dependent intrinsic pathway.

**Figure 2:**
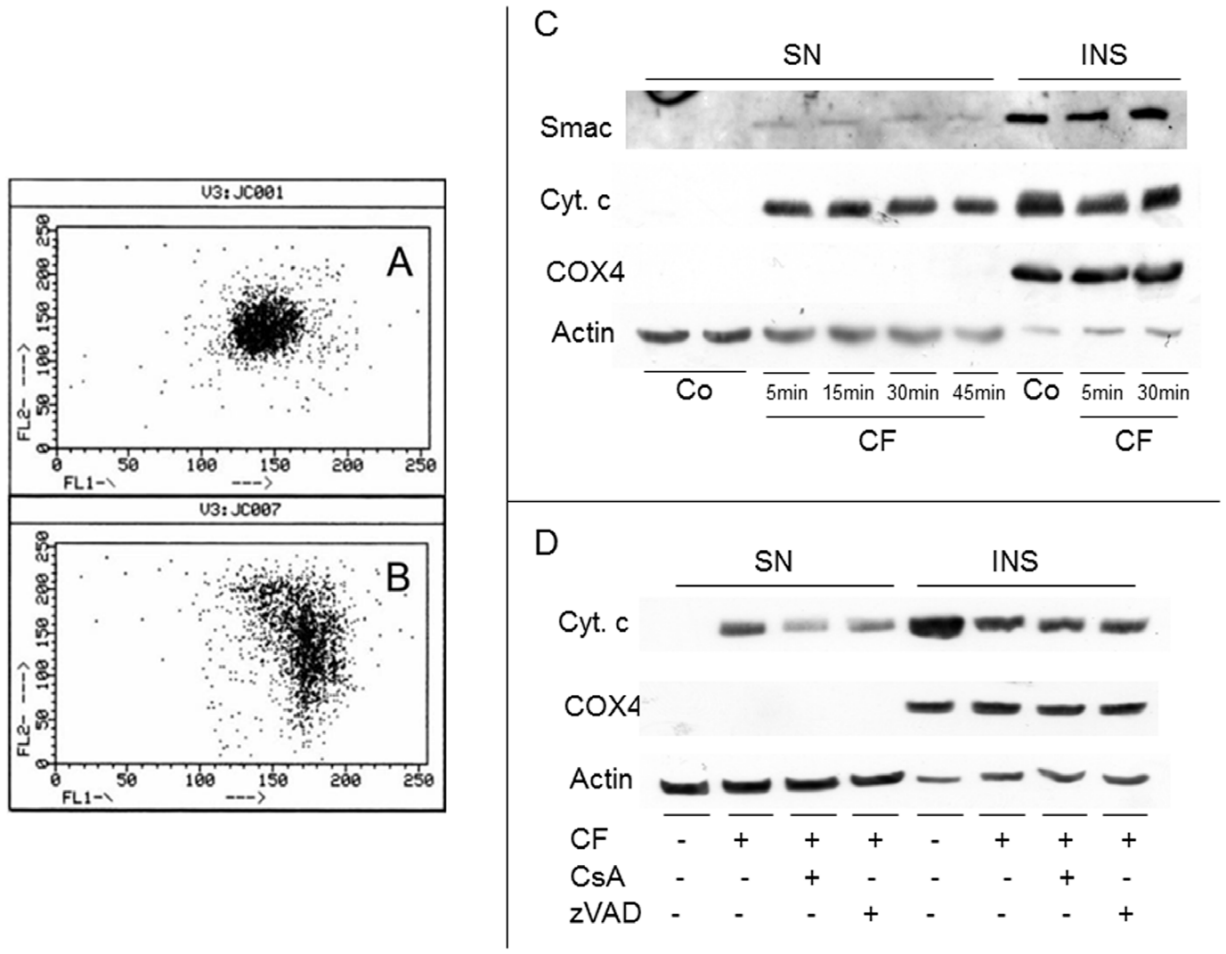
Mitochondrial involvement in clofibrate-induced apoptosis. Shift of the FL2 (590 nm)/FL1 (529 nm; green to red) fluorescence ratio of JC-1 in cells treated with clofibrate (CF) for 5 min (A: control (C); B: CF). (C) representative western blot showing the time course of Smac/DIABLO and cytochrome c appearance in the cytosolic fraction (surnatant, SN). Cytochrome c oxidase subunit IV (COX4) serves as loading control for mitochondrial enriched fraction (insoluble, INS) and actin for cytosolic fraction. (D) western blotting pattern representative of cyclosporine A and zVAD pretreatment on cytochrome c release in cells exposed to CF for 30 min.

The possibility that CF, and fibrate derivatives in general, in addition to the hypolipidemic action, could be exploited also in view of their cytotoxic activity is an interesting one. Indeed, these drugs are currently used in the clinical practice and their side-effects are generally compatible with a good quality of life. In this regard, their possible use in combination with classical antineoplastic treatments is intriguing. The data actually available in the literature are scanty and confusing, showing that CF may be cytotoxic for several tumor cell lines, with poor attention given to the underlying mechanisms. On this line, the present study tries to fill this gap, investigating CF-induced apoptosis in Jurkat cells, a human neoplastic line of hematopoietic origin. The results show that CF quickly activates the apoptotic machinery through a non conventional pathway that at least involves the initiator caspase 2, likely activated via ER stress, and the effector caspase 3, though not caspases 8 and 9.

**Figure 3:**
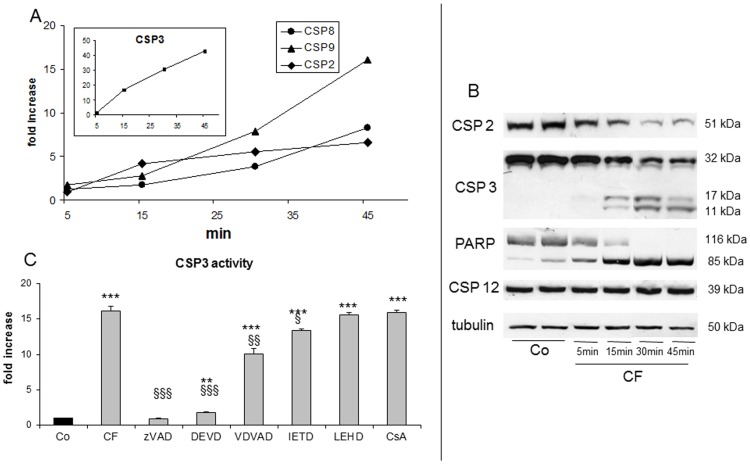
Caspase activation in clofibrate-treated cells. (A) Kinetic of caspase enzymatic activity evaluated by measuring the cleavage of specific fluorogenic substrates. Data are expressed as fold increase respect to control. (B) representative western blotting patterns of caspase and PARP (poly-ADP-ribose polymerase) expression in CF-treated Jurkat cells. Tubulin serves as loading control. (C) Caspase 3 enzymatic activity in cells pretreated with the indicated inhibitors before CF (clofibrate; grey bars) exposure for 30 min. Data (means ± SD, n = 3) are expressed as fold increase respect to Co (control; black bar); ** p<0,01 vs Co; *** p<0,001 vs Co; § p<0,05 vs CF; §§ p<0,01 vs CF; §§§ p<0,001 vs CF.

## Materials and Methods

All materials were supplied by Sigma (St. Louis, MO, USA), unless differently specified.

Jurkat cells (from human leukemic transformed T lymphoma, clone E6-1) were purchased from ATCC (Manassas, VA, USA), seeded in RPMI 1640 medium supplemented with 10% fetal calf serum, 100 U/ml penicillin, 100 mg/ml streptomycin, 2 mM L-glutamine, and maintained at 37°C in a humidified atmosphere of 5% CO_2_ in air. CF, dissolved in DMSO, was added to the medium at the final concentration of 1 mM (or as specified). Controls were treated with the solvent alone. In some experiments Jurkat cells were pretreated with one of the following molecules: zVAD-fmk (zVAD, 1 h, 20 µM), DEVD-cho (DEVD, 1 h, 20 µM), VDVAD-cho (VDVAD, 1 h, 20 µM) LEHD-cho (LEHD, 1 h, 20 µM), IETD-cho (IETD, 1 h, 20 µM) from Alexis Biochemicals (Lausen, Switzerland), cyclosporine A (CsA, 15 min, 1 µM), calpeptin (1 h, 250 µM), bapta-AM (1h, 10 µM) from Biomol (Plymouth Meeting, PA, USA), SP600125 (1 h, 20 µM), PD150606 (1 h, 20 µM), form Calbiochem (La Jolla, Ca, USA) and EGTA (2mM, 30 min).

**Figure 4:**
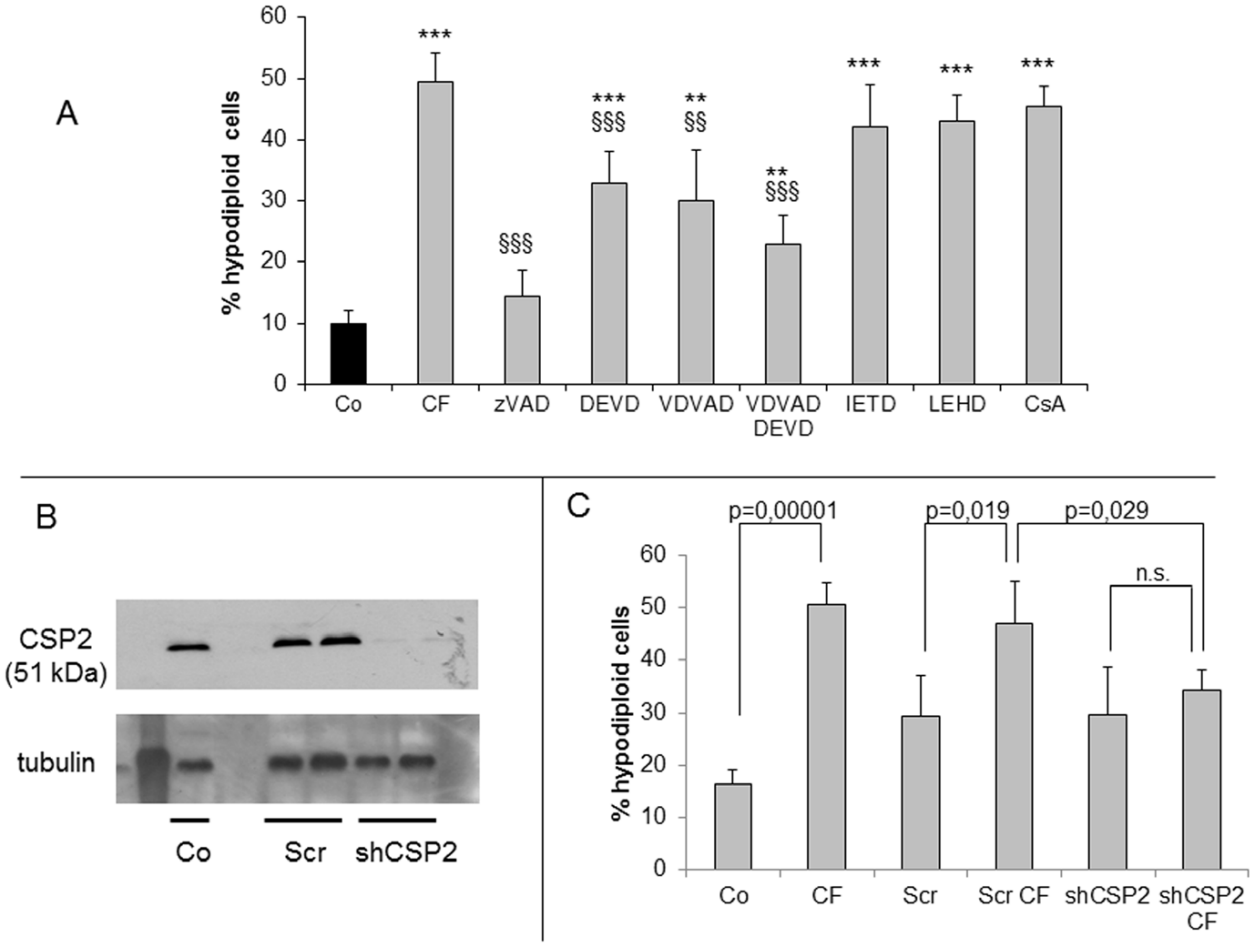
Effect of different caspase inhibitors and caspase 2 silencing on apoptotic death in cells exposed to clofibrate. (A) cells were pretreated with the indicated inhibitors, exposed to clofibrate (CF) and analysed after 45 min. Histograms refer to percentages of cells with hypodiploid DNA content (black bars: untreated; grey bars: CF-treated). (B) sh-RNA effectively abrogates caspase 2 expression in Jurkat cells. Co = control, Scr = scramble shRNA, shCSP2 = caspase 2-specific shRNA; Lane 1: molecular weight marker. Tubulin is used as loading control. (C) flow-cytometric analysis showing the percentages of cells with hypodiploid DNA content of the above mentioned cells exposed to 1 mM clofibrate for 45 min. Data are expressed as mean ± SD (n = 3). ** p<0,01 vs Co; *** p<0,001 vs Co; §§ p<0,01 vs CF; §§§ p<0,001 vs CF.

### Flow Cytometry

DNA distribution analysis was performed as described elsewhere [26]. Briefly, cells were washed in PBS, fixed in ice-cold 70% ethanol for at least 30 min, incubated at room temperature in PBS containing DNase-free RNase (Type II-A) and propidium iodide at the final concentrations of 0.4 mg/ml and 10 µg/ml, respectively. Cells were then analyzed with a FACScan flow cytometer (Becton & Dickinson, Mountain View, CA, USA) equipped with a 488 nm argon laser and three filters, respectively transmitting at 530 nm (FL1), 585 nm (FL2) and above 620 nm (FL3). Data were analysed with the CellQuest software (Becton & Dickinson). The percentage of apoptotic events has been assessed by evaluating the accumulation of cells characterized by a <2n DNA fluorescence.

Mitochondrial depolarization was detected on unfixed cells measuring the fluorescence emission shift of the lipophilic cationic probe 5,5-6,6-tetra-chloro-1,1-3,3-tetraethylbenzimidazolyl-carbocyanine iodide (JC-1, Molecular Probes, Invitrogen Corporation, Carlsbad, CA, USA). JC-1 exhibits potential-dependent accumulation in mitochondria, indicated by a fluorescence emission shift from green (529 nm) to red (590 nm). The loss of mitochondrial membrane potential is indicated by a decreased ratio between red and green fluorescence [27].

Intracellular Ca^2+^ fluctuations were measured using the calcium indicator OregonGreen488-Bapta1-AM (Molecular Probes, Invitrogen Corporation, Carlsbad, CA, USA). Briefly, cells (2⋅10^6^) were loaded with the specific calcium probe (2 µM, 30 min, room temperature) in complete medium without phenol red, centrifuged (600 *g*, 5 min) and resuspended in 1 ml of the same medium. Data capture was performed recording the FL1 mean on 10,000 cells for each time point. CF and/or A23187 were added after reading the baseline fluorescence.

**Figure 5:**
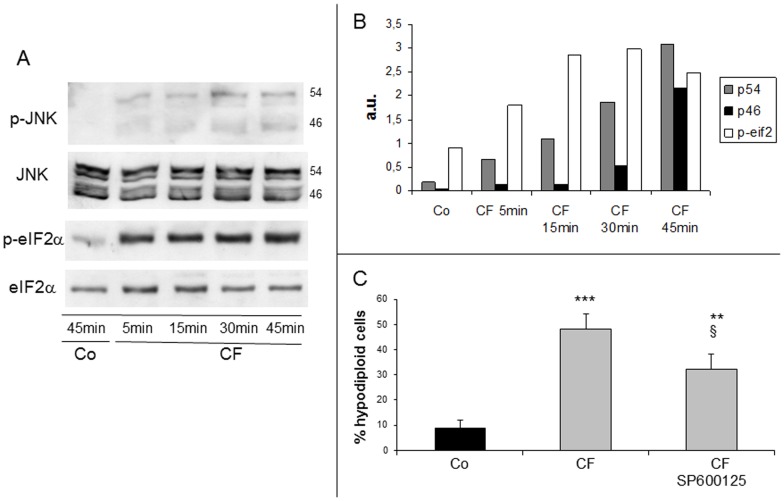
Phosphorylation of eIF2α and JNK activation in clofibrate-treated cells. (A) Representative western blottings of phosphorylated eIF2α and JNK (46 and 54 kDa isoforms). (B) histograms refer to the densitometric analysis. Data are expressed as arbitrary units. (C) Cells were pretreated with the JNK inhibitor SP600125, then exposed to clofibrate (CF) and analysed after 45min. Histograms refer to percentages of cells with hypodiploid DNA content (black bars: untreated; grey bars: CF-treated). Data are expressed as mean ± SD (n = 3). ** p<0,01 vs Co; *** p<0,001 vs Co; § p<0,05 vs CF.

### Caspase Activities

Cells were resuspended in 20 mM HEPES-KOH, pH 7.5, containing 10 mM KCl, 1.5 mM MgCl_2_, 1 mM EDTA, 1 mM EGTA, 1 mM DTT, 1 mM PMSF, frozen and thawed, sonicated, centrifuged (14,000 *g*, 15 min, 4°C), and the supernatant collected. Aliquots corresponding to 20 µg protein were diluted in caspase buffer (25 mM HEPES, pH 7.5, 0.1% CHAPS, 10% sucrose, 10 mM DTT) and assayed for caspase 2, 3, 8, and 9 activities by 1 h incubation at 37°C in the presence of 20 µM substrates (VDVAD-AMC (AMC:7-amino-4-methylcoumarin)), DEVD-AMC, IETD-AMC, LEHD-AMC; Biomol, Plymouth Meeting, PA, USA). The reaction was stopped with 0.1% trichloroacetic acid, and the fluorescence read in a Perkin-Elmer fluorometer (excitation 380 nm – emission 460 nm). Free AMC was used as working standard.

### Western Blotting

Cells were suspended in 0.25% sodium deoxycholate and homogenized by sonication. For cytochrome c detection, cells were suspended in 20 mM HEPES-KOH, pH 7.2, containing 250 mM sucrose, 1 mM EDTA, 0.025% digitonin and freshly added 0.1 mM PMSF, then centrifuged (14,000 *g*, 30 min, 4°C) to obtain the mitochondrial (pellet) and the cytosolic (supernatant) fractions.

Protein concentration was determined by the method of Lowry et al. (1952), using BSA as working standard. Equal amounts of protein (30 µg) were heat-denaturated in sample-loading buffer (50 mM TRIS-HCl, pH 6.8, 100 mM DTT, 2% SDS, 0.1% bromophenol blue, 10% glycerol), resolved by SDS-PAGE and transferred for 2 h to nitrocellulose membranes (Bio-Rad, Hercules, CA, USA). Protein transfer was checked by Ponceau S staining. The filters were then blocked with Tris-buffered saline (TBS) containing 0.05% Tween and 5% non-fat dry milk, and incubated overnight with the following primary antibodies: Smac/DIABLO, poly-ADP-ribose polymerase (PARP), caspase 2, caspase 3, caspase 12, fodrin, JNK (Santa Cruz Biotechnology, Santa Cruz, CA, USA); cytochrome c oxidase subunit IV (COX4), p-eIF2α, eIF2α, p-JNK (Cell Signaling, Danvers, MA, USA); cytochrome c (Becton & Dickinson, Mountain View, CA, USA); actin and tubulin (Sigma, St. Louis, MO, USA). Peroxidase-conjugated IgG (Bio-Rad, Hercules, CA, USA) was used as secondary antibody. The membrane-bound immune complexes were detected by an enhanced chemiluminescence system (Santa Cruz Biotechnology, Santa Cruz, CA, USA) on a photon-sensitive film (Hyperfilm ECL; GE Healthcare, Milano, Italy). Bands were quantified by densitometric scanning of the films and elaborated as described in ‘Data analysis and presentation’.

**Figure 6:**
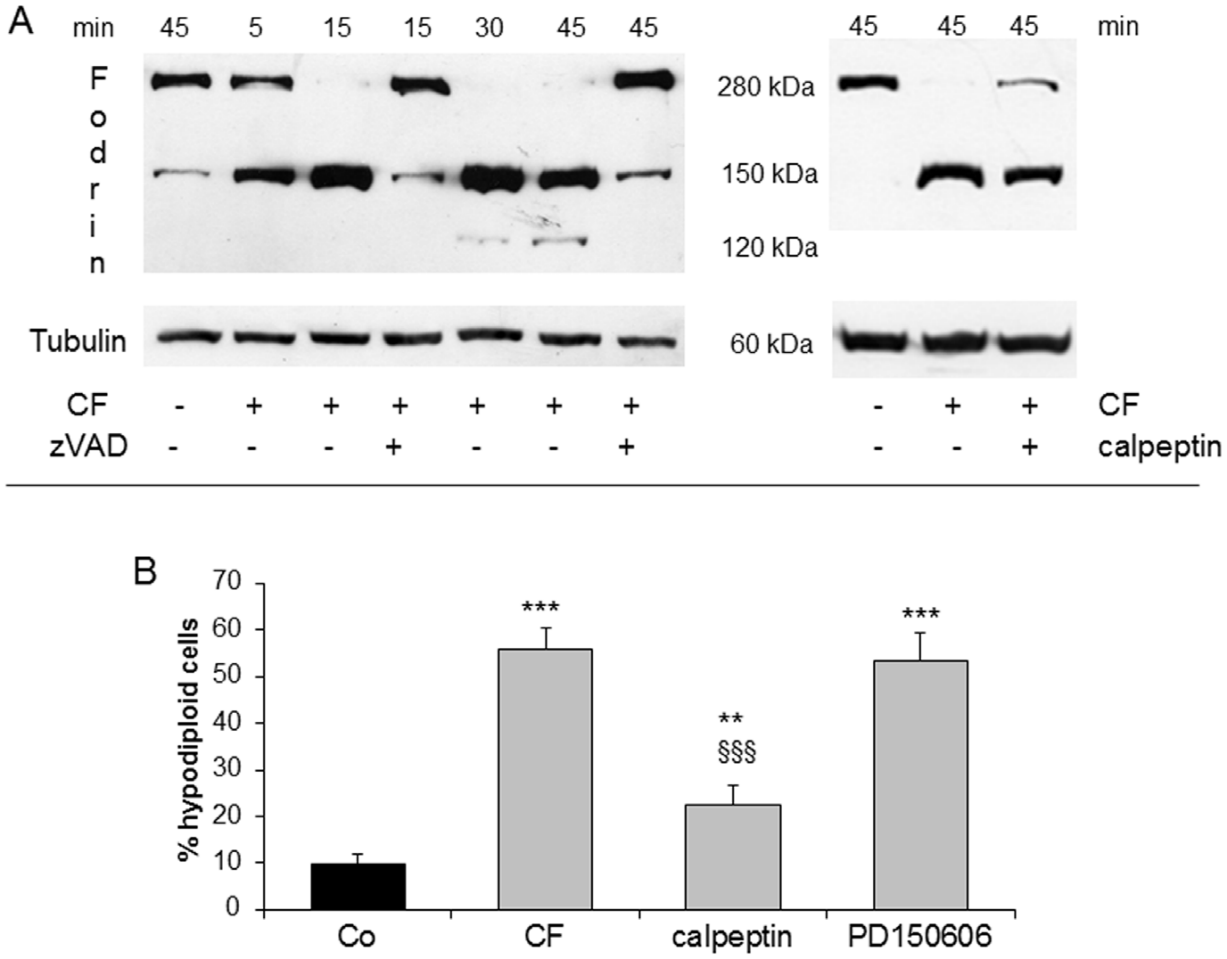
Fodrin cleavage and effect of calpain inhibition in clofibrate-treated cells. (A) Representative western blotting of fodrin cleavage in clofibrate (CF)-exposed Jurkat cells in the presence or in the absence of zVAD and calpeptin. (B) Cells were pretreated with the calpain inhibitors calpeptin or PD150606, then exposed to CF and analysed after 45min. Histograms refer to percentages of cells with hypodiploid DNA content (black bars: untreated; grey bars: CF-treated). Data are expressed as mean ± SD (n = 3). ** p<0,01 vs Co; *** p<0,001 vs Co; §§§ p<0,001 vs CF.

### Caspase-2 Silencing

The shRNA lentiviral vector pLKO.1<-puro bearing the hairpin sequence CCGGGTTGAGCTGTGACTACGACTTCTCGAGAAGTCGTAGTCACAGCTCAACTTTTTG was transfected in packaging HEK 293T cells using calcium phosphate. 24 hours later, the supernatant containing the lentiviral particles was filtered (0,22 µm) and 1 ml added to 10^6^ Jurkat cells. Infected cells were selected adding puromycin 1µg/ml during 2 weeks, tested for silencing efficiency and used for subsequent experiments.

### Data Analysis and Presentation

Results are expressed as means ± standard deviation (SD). Each experiment was performed in triplicate or repeated three times. Image quantification was obtained by densitometry and bands analyzed using a specific software (TotalLab, NonLinear Dynamics, Newcastle upon Tyne, UK). Significance of the differences was evaluated by the Student’s ‘t’ test.

**Figure 7:**
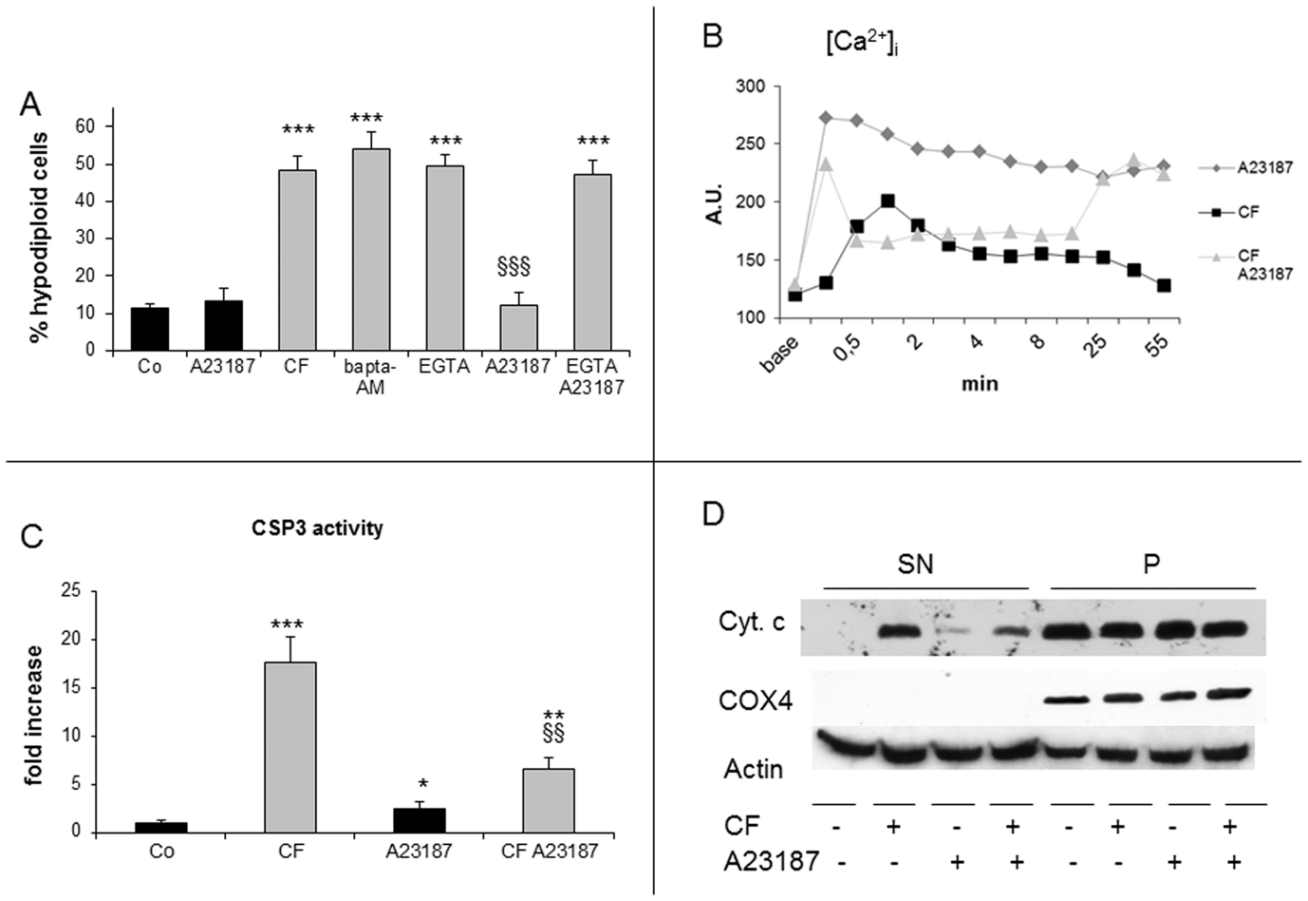
Effect of Ca^2+^ influx on clofibrate-induced apoptosis. (A) Cells were pretreated with OG-Bapta-AM or EGTA, then exposed to clofibrate (CF) and/or the Ca^2+^ ionophor A23187 (5 µM) and analysed after 45min. Data are expressed as mean ± SD (n = 3). *** p<0,001 vs Co; §§§ p<0,001 vs CF. Histograms refer to percentages of cells with hypodiploid DNA content (black bars: without CF; grey bars: CF-treated). (B) cells were incubated with OG-Bapta1-AM and treated with CF and/or A23187. Intracellular calcium release was measured by the increase of 523 nM fluorescence. (C) Caspase 3 enzymatic activity in cells treated with CF and/or A23187 for 30 min. Data (means ± SD, n = 3) are expressed as fold increase respect to control. * p<0,05 vs Co; ** p<0,01 vs Co; *** p<0,001 vs Co; §§ p<0,01 vs CF. (D) western blotting representative of the effect of CF and/or A23187 for 30 min on cytochrome c release. Cytochrome c oxidase subunit IV (COX)4 serves as loading control for mitochondrial enriched fraction (P) and actin for cytosolic fraction (SN).

### Supplemental Material

Primary T cells were isolated using buffy coats from healthy individuals (kindly supplied by CeRMS, Centro Ricerche Medicina Sperimentale, Torino, Italy). PBMCs were separated using the Ficoll-Paque reagent (GE Healthcare, Milano, Italy) following the manufacturer’s instruction. Pan T cells were obtained by negative selection retaining the non-target cells in the magnetic field of a MACS Column (Miltenyi Biotec GmbH, Bergisch Gladbach, Germany) after incubation with a cocktail of biotin-conjugated monoclonal anti-human antibodies against CD14, CD15, CD16, CD19, CD34, CD36, CD56, CD123, and CD235a. The extracted cells were rapidly seeded in complete medium (same as Jurkat cells) and immediately exposed to CF for cytotoxicity assays.

## Results

When exposed to 1 mM CF for 45 min, the standard treatment schedule adopted throughout the present work, approximately 50% of Jurkat cells develop a frankly apoptotic morphology characterized by cell shrinkage due to nuclear and cytoplasmic condensation with nuclear fragmentation (Fig. 1A–B). Comparable effects have been observed on different cell lines, such as human HL-60 promyelocytic leukemia (Fig. S1A) or human CCRF-CEM acute lymphoblastic leukemia (data not shwn), and the murine Colon-26 carcinoma cells (Fig. S1B). Noteworthily, a time/dose study shows that apoptosis is also induced by CF at concentrations lower than 1 mM (Fig. 1C), even if propagating more slowly in the cell population. These concentrations are well within the usual therapeutic (antilipidemic) range that approximates 0.5 mM in patient blood [28]. More pertinently, in pharmacological terms, optimal therapeutic plasma levels of CF have been reported to be around 0.5 mM ([28]; MICROMEDEX Healthcare Series, POISINDEX Managements, Clofibrate and related agents, www.thomsonhc.com). To verify if clofibrate exerts its cytotoxic activity also on normal cells, primary T lymphocytes were exposed to the drug. The results shown in Fig. S2A demonstrate that 45 minutes exposure to 1 mM clofibrate slightly modifies the precentage of hypodiploid cells observed in control cultures. The results do not change when T cells are stimulated with anti-CD3 antibodies for 36 h, in order to have them in a proliferative condition (% of hypodiploid cells: C = 11, CF = 13). In contrast to what observed in Jurkat cells (Fig. 1C), exposure of T lymphocytes to low CF concentrations (0.5 mM) for a period longer than the standard experimental schedule (from 45′ to 8 h) markedly delays the onset of cell death, leading to slightly increased percentage of hypodiploid cells only after 8 h treatment (Fig. S2B).

### Mitochondrial Alterations Induced by CF

A drop in the mitochondrial membrane potential (Fig. 2A–B) as well as a substantial release of cytochrome c to the cytosol (Fig. 2C) manifest rapidly and are already remarkable after a 5 min exposure of Jurkat cells to CF. By contrast, Smac/Diablo is barely detectable in the cytosol (Fig. 2C). The release of cytochrome c to the cytosol is clearly attenuated when the opening of the permeability transition pore is inhibited with cyclosporine A (Fig. 2D) or when cells are pretreated with the polycaspase inhibitor zVAD (Fig. 2D). The latter finding indicates that cytochrome c release is, at least in part, an effect rather then a cause of a proteolytic event inhibited by zVAD.

### CF-induced Death is Caspase-dependent

In a next step, Jurkat cells have been assayed for the enzymatic activity of an effector (3) and three initiator (2, 8 and 9) caspases. As Fig. 3A illustrates, caspase 2-like activity rapidly increases in the first time interval (15 min) and is maintained active afterwards. Conversely, the increase of both caspase 8- and 9-like activities is more evident in the late phases (30–45 min). Finally, the caspase 3-like activity markedly increases in a virtually linear fashion over the whole 45 min interval. Caspase 2 and 3 activation is confirmed by western blotting analysis: the precursor isoforms progressively decrease after exposure to CF, while processed (active) caspase 3 becomes detectable (Fig. 3B), in parallel with an early and extensive cleavage of its endogenous substrate PARP (Fig. 3B). Jurkat cells lack any detectable caspase 4 expression (our original observation, not shown) as well as any caspase 12 activity, which is a general property of human cells [29]. Incidentally, no change in the level of procaspase 12-like immunoreactive protein occurs in CF-treated Jurkat cells over the whole experimental time (Fig. 3B).

CF-induced caspase 3 activation is completely abrogated when cells are pretreated with either polycaspase (zVAD) or caspase 3 (DEVD) inhibitors (Fig. 3C). Among the initiator caspases, only caspase 2 inhibition with VDVAD partially prevents caspase 3 activation, while inhibitors of caspase 8 (IETD) or 9 (LEHD) are ineffective.

As emerging from inhibitor tests, CF-induced cell death is caspase-dependent (Fig. 4A). Cells treated with zVAD become almost completely refractory to CF cytotoxicity. Caspase 2 and 3 inhibitors (respectively, VDVAD and DEVD) afford no more than a partial protection, which becomes higher when they are used concomitantly. Finally, inhibitors such as IETD (caspase 8) or LEHD (caspase 9) do not rescue cells from apoptosis, which is consistent with their inability to affect caspase 3 activation (Fig. 3B). In keeping with this finding, cyclosporine A, which is known to suppress mitochondrial membrane permeability transitions, affords no protection from cell death (Fig. 4A). To clarify if caspase 2 activation is crucial to CF citotoxicity, its expression in Jurkat cells was abrogated by means of shRNA (Fig. 4B). The lack of caspase 2 rendered Jurkat cells more, although not completely, resistant to CF-induced apoptosis. Indeed, the percentage of hypodiploid cells was more than 50% in wild-type cultures, and about 30% in caspase 2-depleted cells (Fig. 4C).

### CF Induces ER Stress

At present, caspase 2 is not univocally associated with a precise cell death pathway, yet there is evidence that on ER stress it may act upstream of mitochondria to promote cytochrome c release [30], [31]. Therefore, we assessed the occurrence of ER stress in CF-treated Jurkat cells by examining the phosphorylation status of the stress-activated SAPK/JNK (46 and 54 kDa isoforms) and of the translation inhibitory factor eIF2α and in both cases observed its progressive increase (Fig. 5A–B). Furthermore, apoptosis by CF is partially prevented in cultures pretreated with the JNK inhibitor SP600125, which suggests a role for JNK in this death process (Fig. 5C).

### CF-induced Apoptosis does not Rely on Calpain Activation

ER stress is often associated with or elicited by Ca^2+^ mobilization from ER stores, which results in increased cytosolic Ca^2+^ concentrations ([Ca^2+^]_i_) that may trigger activation of the Ca^2+^-dependent calpain system [32]. A physiological substrate of the latter is fodrin, a cortical cytoskeletal protein, whose cleavage can thus denote an ongoing calpain activation [33] and has implications for membrane blebbing and phosphatidylserine externalization during apoptosis [34]. In CF-treated Jurkat cells, fodrin cleavage into a 150 kDa product is already prominent at 5 min and virtually complete at 15 min, while a 120 kDa fragment appears after 30 min (Fig. 6A). The 120 kDa fragment is generated by caspase 3 only, whereas both calpain and caspase 3 can cut a similar though not identical 150 kDa fragment [33]. We thus used appropriate inhibitors in order to discriminate between the latter two possibilities. As Fig. 6A illustrates, the 150 and 120 kDa products of fodrin cleavage both nearly disappear in the presence of zVAD, whereas the calpain inhibitor calpeptin affords no more than a weak decrease of the 150 kDa fragment. In apparent contrast with the latter finding, calpeptin markedly protects Jurkat cells from death by CF, yet such protection in not provided at all by another calpain inhibitor such as PD150606 (Fig. 6B). Altogether, these observations suggest that calpains make no significant contribution to CF-induced apoptosis in Jurkat cells.

### Ca2+ Influx Prevents CF-induced Apoptosis

Finally, we tested the hypothesis that altered Ca^2+^ homeostasis could be critical for CF cytotoxicity, even if not necessarily through calpain activation. CF was previously shown [35] to induce transient increases of the [Ca^2+^]_i_ in Jurkat cells. The relevance of such increase to CF-induced apoptosis was not investigated, however. In the present work, neither intracellular nor extracellular Ca^2+^ chelators, such as OG-BAPTA-AM and EGTA, respectively, prevent CF-induced apoptosis (Fig. 7A). In addition, the Ca^2+^ ionophore A23187 by itself exerts no appreciable toxicity on Jurkat cells, at least over the 45 min interval herewith adopted (Fig. 7A). This observation implyes that any change of [Ca^2+^]_i_ caused by CF or the Ca^2+^ influx induced by A23187 separately are well tolerated by these cells. Surprisingly, however, CF cytotoxicity is fully antagonized by A23187 and such protection is abrogated when cells are in the presence of EGTA (Fig. 7A). Not only enforced Ca^2+^ influx is required for cell survival, but also other CF effects such as caspase 3 activation and cytochrome c release are reduced by A23187 (Fig. 7C–D). In agreement with [35], we found CF to induce an early increase of [Ca^2+^]_i,_ peaking at 1 min and followed by a progressive decline (Fig. 7B). A23187 causes a prompt [Ca^2+^]_i_ rise that is largely maintained till the end of the assay. Finally, cotreatment with CF and A23187 results in a complex pattern including an initial [Ca^2+^]_i_ spike followed by a decline to a suprabasal plateau and by an eventual rise to levels close to those produced by A23187, but quite higher than those caused by CF *per se*. These findings can be likely accounted for by the notion [35] that in Jurkat cells CF ≥0.5 mM, not only stimulates a transient [Ca^2+^]_i_ increase, but also inhibits the external Ca^2+^ influx. They also seem to support the view that a [Ca^2+^]_i_ rise neither plays a crucial role in CF-induced apoptosis of Jurkat cells nor is adequate to support calpain activation and fodrin clevage, as shown above.

## Discussion

In recent years, we have been investigating what initially appeared as an unexpected property of CF, namely, its ability to elicit a fast and extensive apoptotic process in cell lines of neoplastic origin, particularly hepatocarcinomas [13], [19], [25], [26], among which the rat Yoshida ascites hepatoma AH-130 provided the main paradigm. The observation that an important lethal action is exerted by CF as well as other PPs on cells of both rodent and human origin prompted us to evaluate carefully these agents for their possible use as antineoplastic drugs. We noticed that human T-lymphoma Jurkat cells are as susceptible as rat hepatoma AH-130 cells to CF-induced apoptosis. This finding suggests that CF could be useful in the treatment of lymphoid malignancies. In this regard, to resort therapies less aggressive in terms of systemic toxicity and side effects would represent a major advance.

### Mechanisms of CF-induced Apoptosis

Among PPAR ligands, only few studies have investigated the cytotoxic mechanisms of those targeting PPARα. Among the latter, some have been reported to activate apoptotic death in various cell lines (reviewed by [36]). In our experience, PPARα activation is unlikely to play any significant role in CF-induced apoptosis of Jurkat (this report) or hepatoma cells, particularly in view of its very rapid time-course [25]. All the potentially relevant parameters herewith evaluated in Jurkat cells are already altered within 5 min exposure to CF, and [Ca^2+^]_i_ within 1 min. Therefore, this fast apoptotic death should be likely categorized as an extrareceptor activity, not differently from other effects of PPARα ligands, such as ER or oxidative stress [12].

In both human Jurkat T-leukemia cells and rat AH-130 hepatoma cells [13], [19], [25], [26] CF-induced apoptosis is caspase-dependent, yet the two death processes follow different courses. As previously found, caspases 3, 8, and 9 are rapidly activated in AH-130 cells exposed to CF and relevant caspase inhibitors afford a significant protection from apoptosis [26]. At variance, we presently found that caspases 8 and 9 activation in Jurkat cells is delayed, particularly with respect to caspase 3 and, in less striking manner, to caspase 2. Moreover, while the mitochondrial pathway appears to play a significant role in CF-treated AH-130 cells [26], in Jurkat cells the pattern is quite different. Briefly, the mitochondrial membrane potential rapidly falls and cytochrome c is released in both cell lines, once exposed to CF, but Smac-Diablo is released from mitochondria in AH-130 cells only (unpublished data). Moreover, caspase-9 inhibition or treatment with cyclosporine A are completely ineffective in protecting Jurkat cells from death. Altogether, these observations suggest that the mithocondrial pathway only marginally contributes to CF-induced apoptosis of Jurkat cells.

The occurrence of ER stress in Jurkat cells exposed to CF is documented by the early and marked increased phosphorylation of two typical targets, eIF2α and JNK. Moreover, a selective JNK inhibitor (SP600125) affords a partial protection from CF-induced apoptosis, suggesting a role for JNK activation in this death. In last years, ER stress has been proposed to trigger apoptosis through mechanisms involving either caspase 12 [37] or 4 or 2, apart from other initiator (8, 9) and effector (3, 7) caspases [38]. Since we ruled out an involvement of both caspases 12 and 4 (see Results), our attention was focused onto caspase 2, which is regarded as an initiator caspase [39]. Dimerization and autocleavage of the p51 caspase 2 precursor lead to formation of an active p37 complex that is subsequently cleaved by an unknown caspase into an inactive 19 kDa fragment [39]. In the present study, the p51 precursor is already decreased in Jurkat cells after 5 min on CF, indicating early activation of caspase 2 that subsequently further increase through the whole experimental time. Of interest, the inactive p19 fragment becomes detectable after 30 min on CF (data not shown), suggesting that a late inhibitory cleavage might maintain constant levels of active caspase 2.

Interestingly, CF has been reported to cause retrograde movement of Golgi constituents to the ER and, independently from PPARα activation, to disrupt the morphological and functional integrity of the Golgi complex in a manner similar to brefeldin A [40], [41]. In conclusion, these observations are compatible with the hypothesis that activation of Golgi- or ER-associated caspase 2 are involved in the activation of caspase 3 in CF-treated cells.

### Role of Intracellular [Ca^2+^] in CF Cytotoxicity

Both ER stress and mitochondrial depolarization may result in increases of cytosolic [Ca^2+^]_ i_, which should be expected to activate calpains. The latter in turn can be involved in the early phases of apoptosis, leading to cleavage of the effector caspase 3 [42], [43], [44]. As an example, coincubation of Jurkat cells with *Entamoeba histolytica* results in calpain-dependent caspase 3 activation and apoptosis, that can be prevented by calpeptin [45]. However, the involvement of calpain activation in CF-induced cell death appears unlikely, in view of two different observations: (i) even if calpeptin partially protects Jurkat cells from CF, this effect probably relies on non-specific caspase inhibition, since calpeptin only barely attenuates the intracellular fodrin cleavage if compared to the polycaspase inhibitor zVAD; (ii) no significant protection from CF is afforded by PD150606, another calpain inhibitor. A [Ca^2+^]_i_ rise can result either in pro- or antiapoptotic effects [46]. In the present work, a [Ca^2+^]_i_ elevation occurs after CF, yet treatments aimed at inhibiting it by means of extra- or intracellular chelators confer no protection against CF-induced cell death. By contrast, Ca^2+^ influx elicited by A23187 unexpectedly exerts a marked antiapoptotic action in CF-treated Jurkat cells, this protection being abrogated by adding EGTA into the medium. Therefore, protection by A23187 appears to depend on its ability to increase the Ca^2+^ influx in CF-treated cells, where it also inhibits both cytochrome c release and caspase 3 activation. The present data match a previous report of an antiapoptotic action of Ca^2+^ influx in puromycin-treated U937 cells [46]. The negligible toxicity of A23187 itself on Jurkat cells remains to be clarified. Noteworthily, T-cells tolerate high [Ca^2+^]_i_ as required for activation, proliferation, and cytokine synthesis (reviewed by [47]), nor death occurs in B-lymphoma cell cultures exposed to anoxia/reoxygenation, in spite of a sustained [Ca^2+^]_i_ rise [48].

### Conclusion

The present study demonstrates that exposing Jurkat cells to CF triggers a caspase-dependent apoptotic process not conforming to a canonical pathway, wherein caspases 2, likely activated by ER stress, and caspase 3 are involved. Finally, both apoptosis and caspase 3 activation are prevented by Ca^2+^ influx, suggesting that mobilization of Ca^2+^ stores from the ER combined with suppression of Ca^2+^ influx are as well a crucial event in the pathway to cell death.

The present work on Jurkat cells significantly improves the knowledge concerning PPARα ligand toxicity on tumor-derived cell lines. So far, the data in the literature are relatively scanty and quite scattered in terms of cell types and drugs investigated, treatment schedule, and kinetic of the events under study. Two groups of neoplastic cell lines received the largest share of attention so far: those belonging to the hepatocytic lineage (see Introduction) and those of hematopoietic origin. Among the latter, evidence for growth arrest, induced differentiation and apoptosis driven by PPARα ligands, alone or combined with other drugs, has been obtained on neoplastic human cells of both lymphoid ([49], [50], [20]) and myeloid origin ([51], [52], [53], [54], [55]). On this basis, we can at least conclude that remarkable antiblastic properties are exhibited by a variety of PPARα ligands. In particular, clinically relevant concentrations of CF exert a strong apoptogenic action on human T-leukemia Jurkat cells, comparable with that on rat hepatoma Yoshida AH-130 cells. Therefore, the present results suggest that CF, a PPAR agonist that has been widely used in the clinical handling of hyperlipemias, is worth being further scrutinized as a potential low-toxicity and low-cost drug for leukemia therapy. Further work on this issue is thus advisable.

## Supporting Information

Figure S1 **Clofibrate-induced apoptosis in HL-60 and C26 cells.** Representative plots of flow-cytometric analysis (see Materials and Methods for details). Panel A: HL-60 cells, panel B: C26 colon adenocarcinoma cells. M1 represents the percentage of cells with hypodiploid DNA content (apoptotic).
(TIF)

Figure S2 **Effect of clofibrate on primary T lymphocytes.** Representative plots of flow-cytometric analysis (see Materials and Methods for details), M1 represents the percentage of cells with hypodiploid DNA content (apoptotic). Panel A: T cells exposed to 1 mM CF for 45 min; Panel B: T lymphocytes exposed to 0.5 mM clofibrate at different time points (from 45 min to 8 h).
(TIF)
