# Supplementary figures and images for: Caspase 2 Activation and ER Stress Drive Rapid Jurkat Cell Apoptosis by Clofibrate
Source: PLoS One. 2012 Sep 18;7(9):e45327. doi: 10.1371/journal.pone.0045327 (PMC3445471; doi:10.1371/journal.pone.0045327)

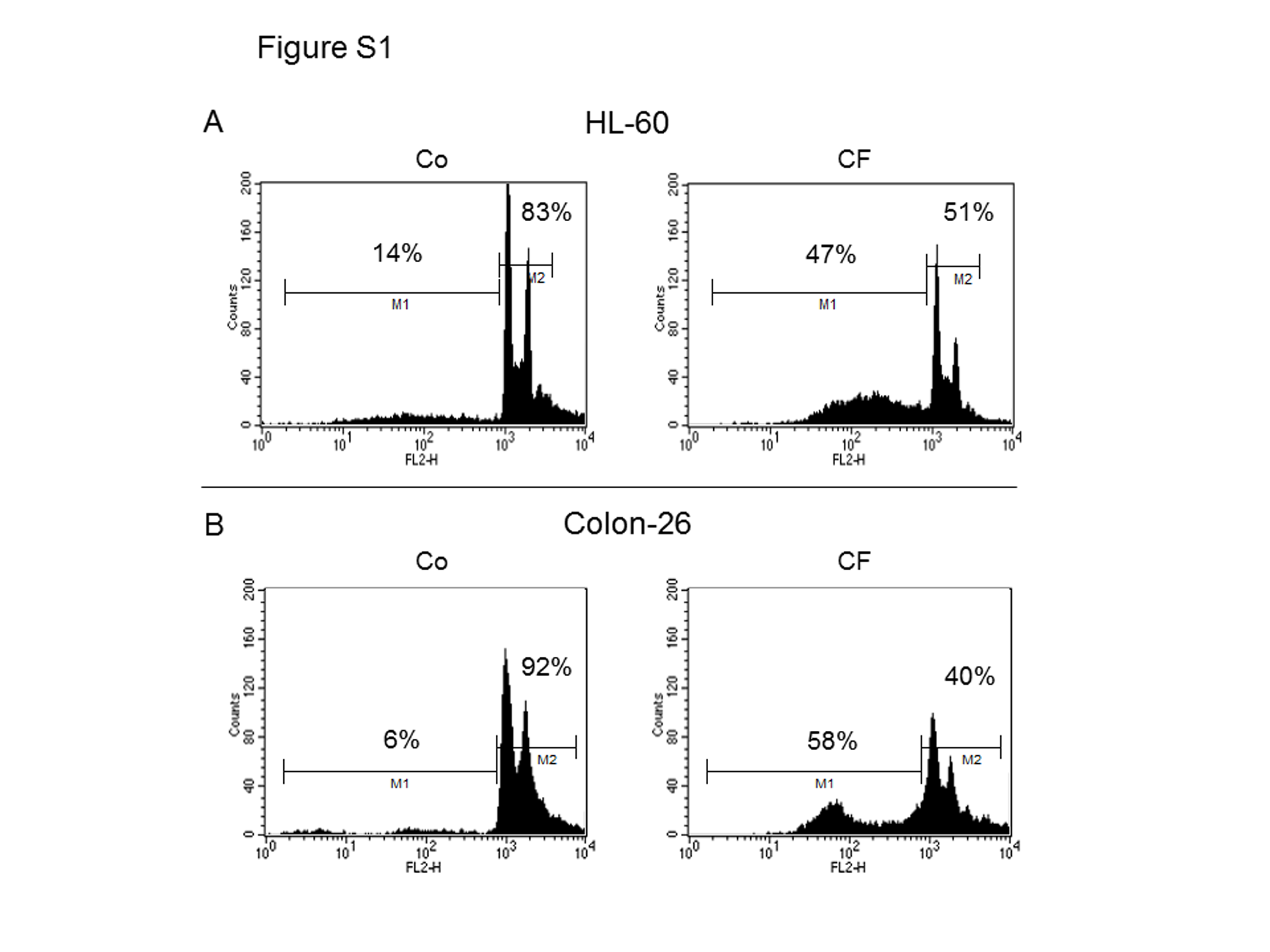

Supplement: Figure S1 — Clofibrate-induced apoptosis in HL-60 and C26 cells. Representative plots of flow-cytometric analysis (see Materials and Methods for details). Panel A: HL-60 cells, panel B: C26 colon adenocarcinoma cells. M1 represents the percentage of cells with hypodiploid DNA content (apoptotic). (TIF) [file pone.0045327.s001.tif]
